# Preoperative CT-based radiomic prognostic index to predict the benefit of postoperative radiotherapy in patients with non-small cell lung cancer: a multicenter study

**DOI:** 10.1186/s40644-024-00707-6

**Published:** 2024-05-13

**Authors:** Zeliang Ma, Yu Men, Yunsong Liu, Yongxing Bao, Qian Liu, Xu Yang, Jianyang Wang, Lei Deng, Yirui Zhai, Nan Bi, Luhua Wang, Zhouguang Hui

**Affiliations:** 1 Department of Radiation Oncology, National Clinical Research Center for Cancer/Cancer Hospital/National Cancer Center, Chinese Academy of Medical Sciences and Peking Union Medical College, Beijing, China; 2 Department of VIP Medical Services, National Clinical Research Center for Cancer/Cancer Hospital/National Cancer Center, Chinese Academy of Medical Sciences and Peking Union Medical College, Beijing, China; 3 Department of Medical Oncology, National Clinical Research Center for Cancer/Cancer Hospital/National Cancer Center, Chinese Academy of Medical Sciences and Peking Union Medical College, Beijing, China

**Keywords:** Radiomics, Radiotherapy, Survival, Non-small cell lung cancer, CT

## Abstract

**Background:**

The value of postoperative radiotherapy (PORT) for patients with non-small cell lung cancer (NSCLC) remains controversial. A subset of patients may benefit from PORT. We aimed to identify patients with NSCLC who could benefit from PORT.

**Methods:**

Patients from cohorts 1 and 2 with pathological Tany N2 M0 NSCLC were included, as well as patients with non-metastatic NSCLC from cohorts 3 to 6. The radiomic prognostic index (RPI) was developed using radiomic texture features extracted from the primary lung nodule in preoperative chest CT scans in cohort 1 and validated in other cohorts. We employed a least absolute shrinkage and selection operator-Cox regularisation model for data dimension reduction, feature selection, and the construction of the RPI. We created a lymph-radiomic prognostic index (LRPI) by combining RPI and positive lymph node number (PLN). We compared the outcomes of patients who received PORT against those who did not in the subgroups determined by the LRPI.

**Results:**

In total, 228, 1003, 144, 422, 19, and 21 patients were eligible in cohorts 1–6. RPI predicted overall survival (OS) in all six cohorts: cohort 1 (HR = 2.31, 95% CI: 1.18–4.52), cohort 2 (HR = 1.64, 95% CI: 1.26–2.14), cohort 3 (HR = 2.53, 95% CI: 1.45–4.3), cohort 4 (HR = 1.24, 95% CI: 1.01–1.52), cohort 5 (HR = 2.56, 95% CI: 0.73–9.02), cohort 6 (HR = 2.30, 95% CI: 0.53–10.03). LRPI predicted OS (C-index: 0.68, 95% CI: 0.60–0.75) better than the pT stage (C-index: 0.57, 95% CI: 0.50–0.63), pT + PLN (C-index: 0.58, 95% CI: 0.46–0.70), and RPI (C-index: 0.65, 95% CI: 0.54–0.75). The LRPI was used to categorize individuals into three risk groups; patients in the moderate-risk group benefited from PORT (HR = 0.60, 95% CI: 0.40–0.91; *p* = 0.02), while patients in the low-risk and high-risk groups did not.

**Conclusions:**

We developed preoperative CT-based radiomic and lymph-radiomic prognostic indexes capable of predicting OS and the benefits of PORT for patients with NSCLC.

**Supplementary Information:**

The online version contains supplementary material available at 10.1186/s40644-024-00707-6.

## Introduction

The value of postoperative radiotherapy (PORT) for patients with pN2 non-small cell lung cancer (NSCLC) remains controversial [1–4]. Previous retrospective studies demonstrated that PORT was advantageous [5, 6]. In contrast, two recent randomised clinical trials (RCTs) [7, 8] only showed non-significant improvement of disease-free survival (DFS) or overall survival (OS) from PORT for these unselected patients; however, PORT could significantly decrease locoregional recurrence. Additionally, the toxicities of PORT [9] — cardiopulmonary toxicity in particular [7, 10–12] — may diminish its benefit. Owing to these findings, we aimed to predict the prognosis of these patients and identify the individuals who could benefit from PORT. Few studies have identified suitable markers that are valuable for identifying such individuals.

Previous studies have shown that PORT can improve the OS of highly selected patients with NSCLC [13, 14]. However, the study was only based on clinical features, and the results were not validated externally. Computed tomography (CT)-based radiomics consists of quantitative imaging characteristics derived from radiographic images and has recently attracted interest as a potential tool for diagnosis and prognosis prediction in lung cancer [15]. To our knowledge, no study has attempted to use radiomics to identify patients with NSCLC who may benefit from PORT. By accurately determining which patients need PORT, their survival could be significantly prolonged. Simultaneously, identifying patients who do not require PORT allows them to avoid unnecessary side effects and reduce medical costs.

This study aimed to develop and validate a quantitative radiomic prognostic index (RPI) using radiomic features from preoperative CT scans to predict OS in patients with NSCLC in six independent cohorts. Additionally, we developed a lymph-radiomic prognostic index (LRPI) that combined RPI and positive lymph node (PLN) amount to identify patients who would benefit from PORT.

## Methods and materials

### Patients

Six independent cohorts were included in this study. Cohort 1 included participants from the PORT-C RCT (NCT00880971) conducted at China’s National Cancer Center between January 1st, 2009 and December 31st, 2017. Cohort 2 included eligible real-world patients at China’s National Cancer Center between 1 June 2010 and 1 June 2019. The inclusion criteria were patients with histologically proven pN2 NSCLC who underwent complete resection. Patients who underwent pneumonectomy and those with a history of additional malignancies or any neoadjuvant treatment were excluded. Patients with available preoperative diagnostic CT scans and survival and recurrence data were enrolled. Patients who died or experienced recurrence within six months after surgery were excluded to minimise immortal time bias because the four cycles of chemotherapy and PORT took about six months. Patients were divided into the PORT group and the non-PORT group. Non-PORT group in cohort 1 was used to establish the RPI and LRPI. Real-world validation was performed in cohort 2.

Cohorts 3–6 served as external validation sets. Cohort 3 comprised patients with early-stage NSCLC who underwent surgical treatment between 7 April 2008 and 15 September 2012 at the Stanford University School of Medicine and Palo Alto Veterans Affairs Healthcare System, USA [16]. Cohort 4 enrolled patients treated at MAASTRO Clinic, The Netherlands, with inoperable, histologic or cytologic confirmed NSCLC, Union for International Cancer Control (UICC) stages I-IIIb, treated with radical radiotherapy alone (*n* = 196) or with chemo-radiation (*n* = 226) [17]. Cohorts 5 and 6 enrolled patients from The Cancer Genome Atlas Lung Adenocarcinoma (TCGA-LUAD) [18] and Lung Squamous Cell Carcinoma (TCGA-LUSC) data collection [19].

### Procedures

Pyradiomics (v3.0.1) was used to extract radiomic features from chest CT images. Previous studies have shown that the convolution kernel significantly affects feature extraction [20]; thus, for cohort 1 and 2, we limited the convolution kernel to include STANDARD, B31s, and FC14. For Cohorts 3 through 6, we intentionally did not restrict the CT acquisition parameter to evaluate the robustness of the model across varied imaging settings. We used a uniform slice thickness of 5 mm for all cohorts. Each primary tumour was manually contoured in the axial view by two experienced radiation oncologists using MIM software (MIM, 7.1.4). From each annotated area, 1,409 radiomic features from seven distinct feature families (shape, first-order, grey-level co-occurrence matrix, grey-level dependence matrix, grey-level size zone matrix, grey-level run-length matrix, and neighbouring grey-tone difference matrix) were retrieved. All features were normalised with max-minimum normalisation in each cohort. The most predictive features for the non-PORT group in cohort 1 were used to develop the RPI. The formula for calculating the RPI can be found in the supplementary materials. An elevated RPI indicates an increased mortality risk.

### Transcriptomic analysis and pathological evaluation

We calculated the tumour stromal ratio (TSR) with QuPath [21] from the surgically resected whole-slide tissue scans of 206 patients in cohort 2 to determine the histomorphometry correlation with the RPI. We examined the relationship between radiomic features and TSR. Detailed methods to calculate TSR are available in the supplements. To understand the biological mechanism of RPI, raw and processed RNA sequencing data were collected for 130 subjects in cohort 3, available on the NCBI Gene Expression Omnibus (GEO) [21]. 117 patients with corresponding CT scans and segmentations were selected for transcriptomic analysis. We employed GEO2R for the differential gene expression analysis and displayed the result as a volcano plot. Subsequently, we implemented Gene Set Enrichment Analysis (GSEA) over the Reactome database using the ReactomePA R package (v1.44.0), and Kyoto Encyclopedia of Genes and Genomes (KEGG) GSEA using the clusterProfiler R package (v4.8.1) to identify pertinent biological pathways.

### Statistics

Continuous variables are presented as mean ± standard deviation for normally distributed data and median ± interquartile range (IQR) for non-normally distributed data. Normality was checked using the Shapiro-Wilk test. Categorical variables are presented as count and percentage. Continuous variables were compared using one-way ANOVA or Kruskal-Wallis test; Categorical variables were compared using χ2 test. This study applied the least absolute shrinkage and selection operator (LASSO), an appropriate technique for high-dimensional data, to select the most relevant variables for OS among the non-PORT group in cohort 1. A detailed explanation and calculation of the selected radiomic features can be found in the supplement methods. LASSO coefficients were used to create the RPI for each patient using a linear combination of the chosen radiomic features and their corresponding coefficients as weights. The value of the tuning parameter in the LASSO-Cox model was averaged across 10 cross-validations to reduce error. Significant clinicopathological risk variables were identified using Cox regression analysis, which were combined with the RPI to build an LRPI.

For prognostic stratification, the median RPI values were used to separate cohort 1 into two groups, for which the OSs and hazard ratios (HRs) were determined. Kaplan–Meier survival analysis, the log-rank test, and HR were used to confirm the predictive performance of the RPI. The smoothHR package was utilised to illustrate the increase in mortality risk as the RPI escalates. LRPI was used to split patients into three risk groups to identify patients who would benefit from PORT. Version 4.1.0 of R was used for statistical analyses.

## Results

A total of 228 patients were eligible for inclusion in cohort 1, of whom 96 (42%) were in the PORT group, and 132 (58%) were in the non-PORT group. In cohort 2, 1,003 eligible patients were identified for real-world validation; 227 (23%) were in the PORT group, and 776 (77%) were in the non-PORT group. A total of 144 patients were eligible in cohort 3. The baseline characteristics of the patients are presented in Table 1. Baseline information of cohort 4–6 was not available. Moreover, cohorts 4, 5, and 6 consisted of 422, 19, and 21 patients, respectively. Three unique radiomic features (Supplementary Fig. 1) were selected using LASSO-Cox analysis to generate the RPI. The RPI distribution across these six cohorts were shown in sFigure 2.

**Table 1:** Patient characteristics

Characteristic	Level	Cohort 1	Cohort 2	Cohort 3	*p*
n		228	1003	144	
Sex (%)	Male	122 (53.51)	566 (56.43)	108 (75.00)	< 0.01
	Female	106 (46.49)	437 (43.57)	36 (25.00)	
Age (median [IQR])		55.00 [49.00, 60.00]	59.00 [52.00, 65.00]	69.00 [64.00, 76.00]	< 0.01
KPS (median [IQR])		90.00 [80.00, 90.00]	90.00 [90.00, 90.00]	Not available	< 0.01
Smoking history	Absence	129 (56.58)	530 (52.84)	22 (15.28)	< 0.01
	Presence	99 (43.42)	473 (47.16)	122 (84.72)	
Tumor location	Left lung	92 (40.35)	457 (45.56)	58 (40.28)	0.22
	Right lung	136 (59.65)	546 (54.44)	86 (59.72)	
Histology	non-SCC	198 (86.84)	824 (82.32)	115 (79.86)	0.16
	SCC	30 (13.16)	177 (17.68)	29 (20.14)	
pT (%)	T1	49 (21.49)	198 (19.74)	74 (51.39)	< 0.01
	T2-3	179 (78.51)	805 (80.26)	70 (48.61)	
PLN (median [IQR])		4.00 [2.00, 7.00]	5.00 [2.00, 9.00]	Not available	< 0.01
PORT (%)	NO	132 (57.89)	776 (77.37)	132 (91.67)	< 0.01
	YES	96 (42.11)	227 (22.63)	12 (8.33)	

*Abbreviation* KPS, Karnofsky performances status; SCC, squamous cell carcinoma; PLN: positive lymph node; IQR, interquartile range; PORT: postoperative radiotherapy

**Fig. 1:**
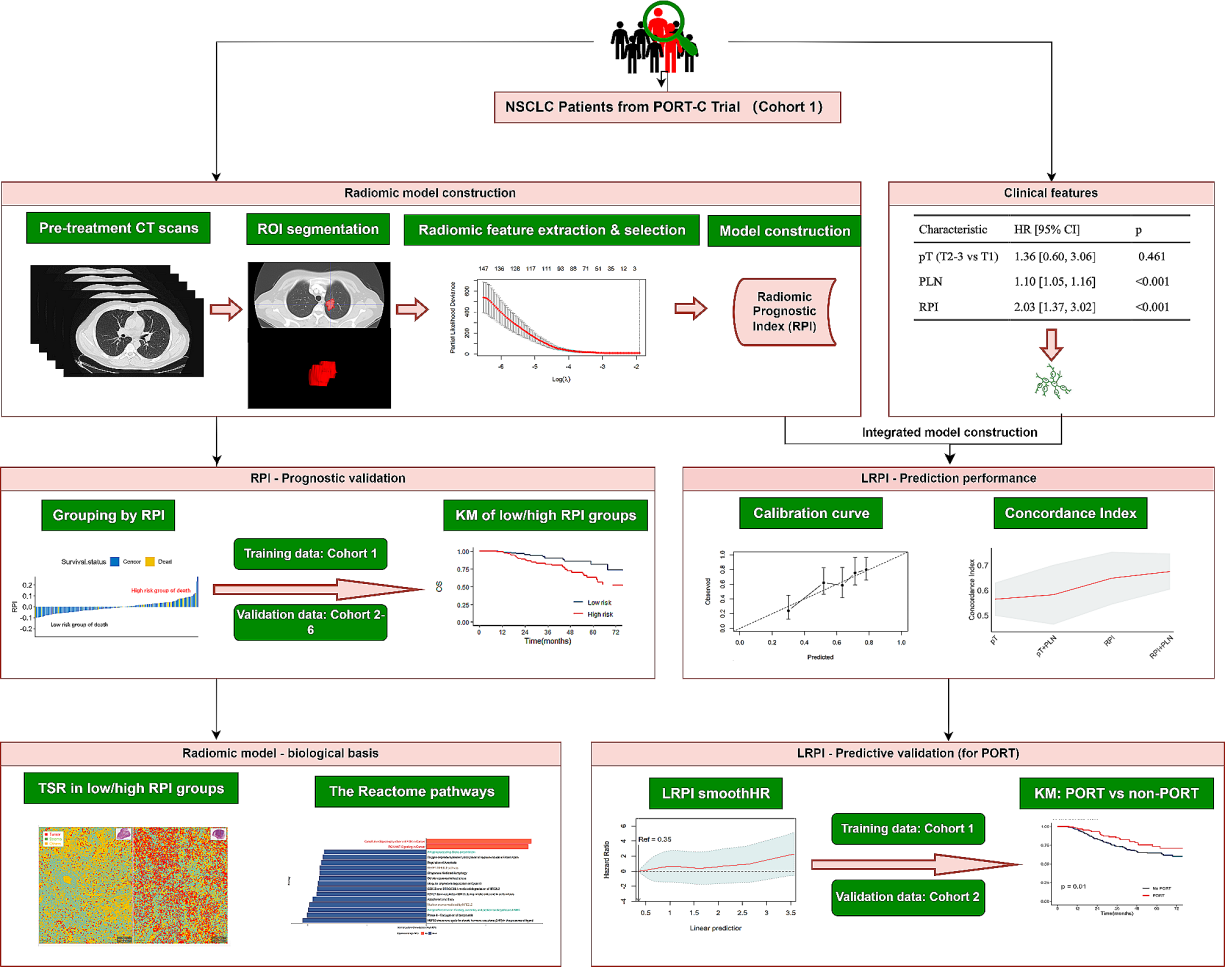
Comprehensive outline and pipeline of the study.The initial step encompassed the identification and annotation of the primary nodule evident in the CT scan. Pyradiomics was employed to extract intra-tumoural textural features. Using the LASSO-Cox method, top features were selected and applied in the construction of RPI. The LRPI was constructed utilising prognostic clinical features alongside RPI. The prognostic performance of RPI and the predictive potential of LRPI for the benefit of PORT were subsequently validated. Furthermore, the relationships between RPI features and the tumour-stroma ratio, as seen on whole slide imaging, were evaluated. Correlations between RPI and mRNA data were also explored to investigate the underlying biological pathways. LASSO, least absolute shrinkage and selection operator; RPI, radiomic prognostic index; LRPI, lymph-radiomic prognostic index; PORT, postoperative radiotherapy

**Fig. 2:**
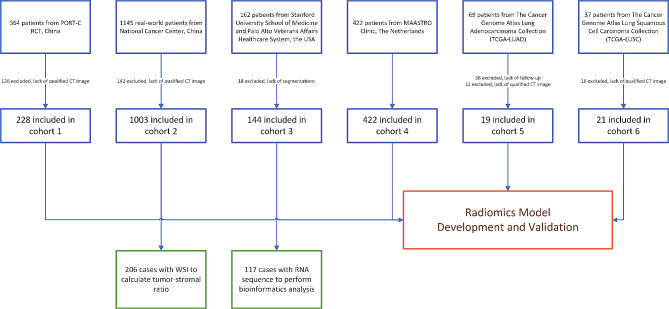
Study profile

**Fig. 3:**
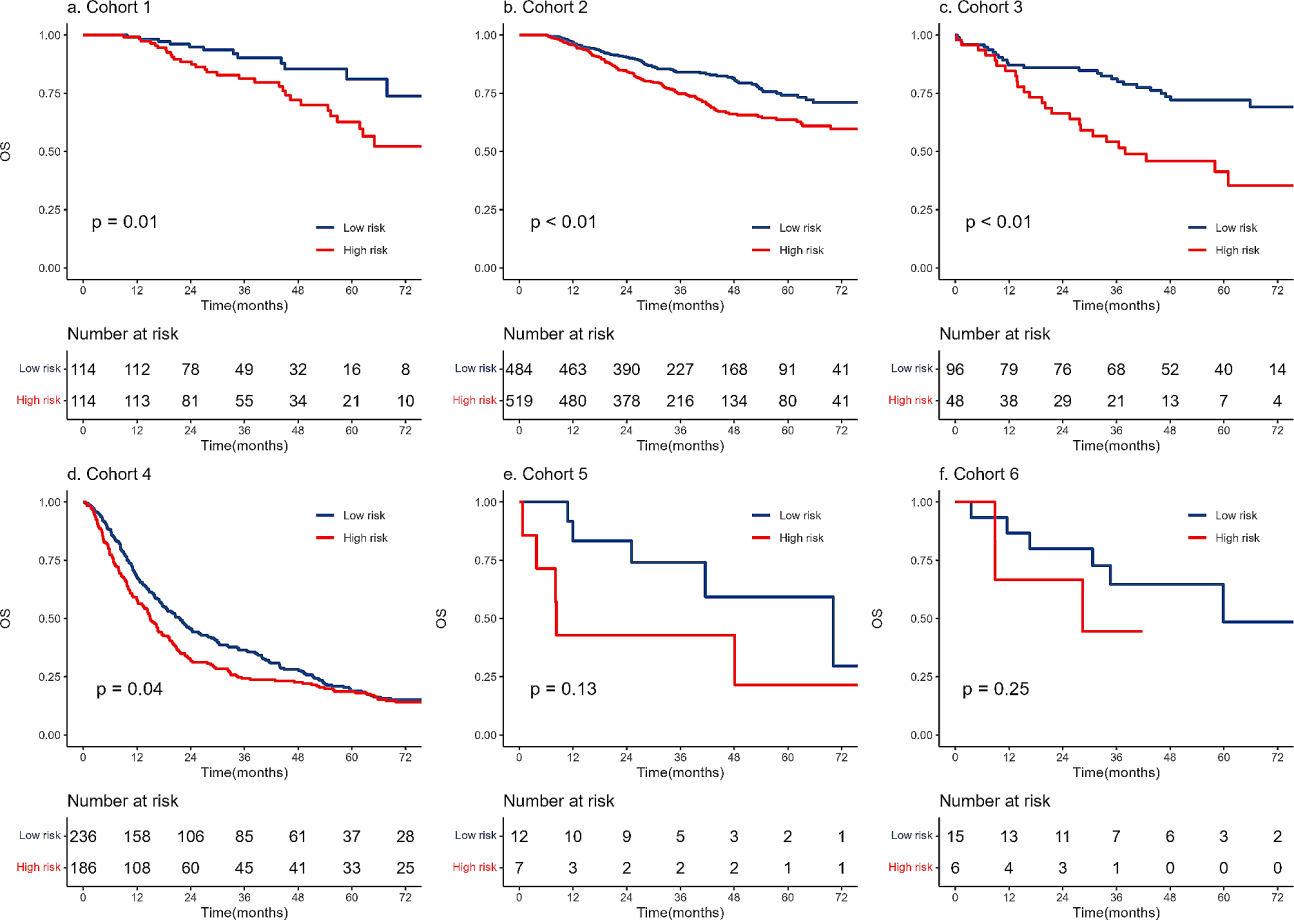
Kaplan-Meier curve for overall survival between RPI-based risk groups. The RPI was developed in cohort 1 and validated in cohorts 2–6. RPI, radiomic prognostic index

Based on the median RPI of -0·00758, two groups with high and low risks were found. RPI predicted OS in all cohorts: cohort 1 (HR = 2·31, 95% CI 1·18–4·52, *p* = 0·01), cohort 2 (HR = 1·64, 95% CI 1·26–2·14, *p* < 0·01), cohort 3 (HR = 2·53, 95% CI 1·45–4·39, *p* < 0·01), cohort 4 (HR = 1·24, 95% CI 1·01–1·52, *p* = 0·04), cohort 5 (HR = 2·56, 95% CI 0·73–9·02, *p* = 0·14), cohort 6 (HR = 2·30, 95% CI 0·53–10·03, *p* = 0·27). Patients with low RPIs had enhanced OS, whereas patients with high RPIs had reduced OS (Figs. 1, 2 and 3).

**Fig. 4:**
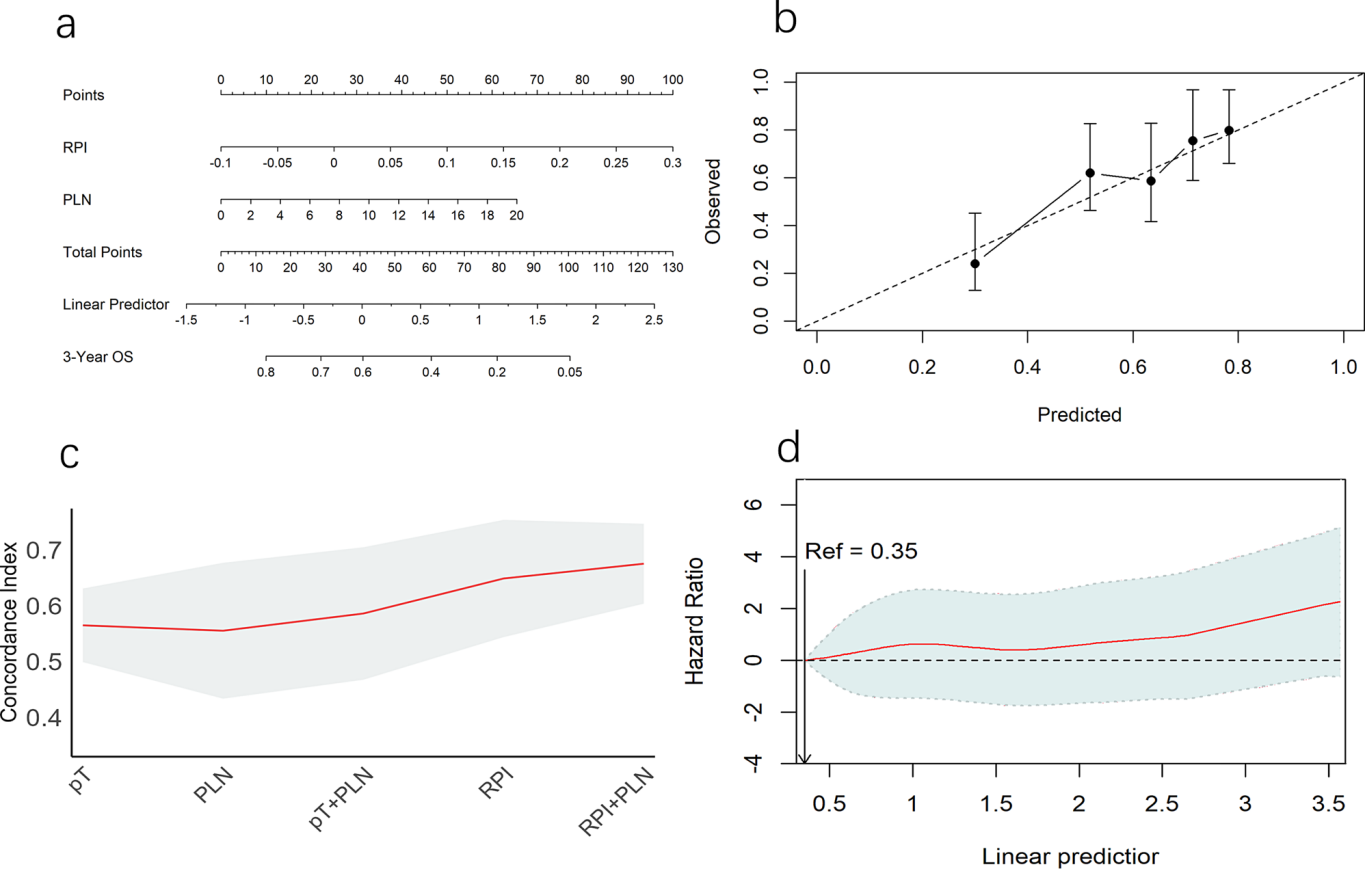
LRPI for predicting overall survival. **a** LRPI represented as nomogram. **b** Calibration curve showcasing the prognostic performance of LRPI. **c** Concordance index demonstrating the comparison between LRPI and prognostic clinical features. **d** Calculated logarithm of hazard ratios (solid lines), along with the 95% confidence intervals (shaded areas) for the association between LRPI and overall survival. LRPI, lymph-Radiomic prognostic index

Univariate analysis of clinical variables in cohort 1 revealed that pathologic tumour stage (pT), positive lymph node number (PLN) were prognostic factors of OS (Supplementary Table 1). However, in the multivariate analysis, pT were no longer correlated with OS, whereas PLN and RPI remained significant predictors (Supplementary Table 2). Using PLN and the RPI, we created an LRPI to predict the 3-year OS, depicted as a nomogram (Fig. 4a). The predicted and observed OS rates were comparable, as evidenced by the Hosmer–Lemeshow calibration curve (Fig. 4b). The LRPI predicted OS (C index 0·68, 95% CI 0·60–0·75) better than the pT (C index 0·57, 95% CI 0·50–0·63), pT + PLN (C index 0·58, 95% CI 0·46–0·70), and RPI (C index 0·65, 95% CI 0·54–0·75).

(Fig. 4c). A plateau was observed in the smoothHR curve between LRPI values of 1.5 and 2.3 (Fig. 4d) in the PORT group of cohort 1. We utilized these two points to categorize patients into low, moderate, and high-risk groups.

**Fig. 5:**
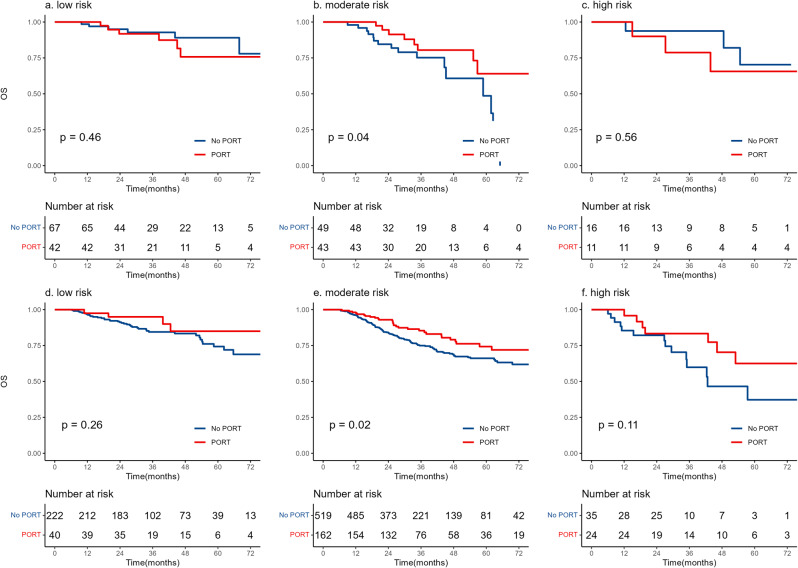
Overall survival between PORT and non-PORT in LRPI-based risk subgroups. Patients in the low-risk and high-risk groups did not benefit from PORT, while patients in the moderate-risk group did. PORT, postoperative radiotherapy; LRPI, lymph-radiomic prognostic index

The LRPI was used to categorise individuals to identify those who would predict the benefit of PORT. In cohort 1, patients in the low-risk group (HR = 0·57, 95% CI 0·23–1·44, *p* = 0·23, Fig. 5a) and high-risk group (HR = 0·55, 95% CI 0·22–1·39, *p* = 0·21, Fig. 5c) did not benefit from PORT, while patients in moderate risk group did (HR = 0·60, 95% CI 0·40–0·91, *p* = 0·02, Fig. 5b). Consistent results were found in cohort 2 (Fig. 5d-f).

**Fig. 6:**
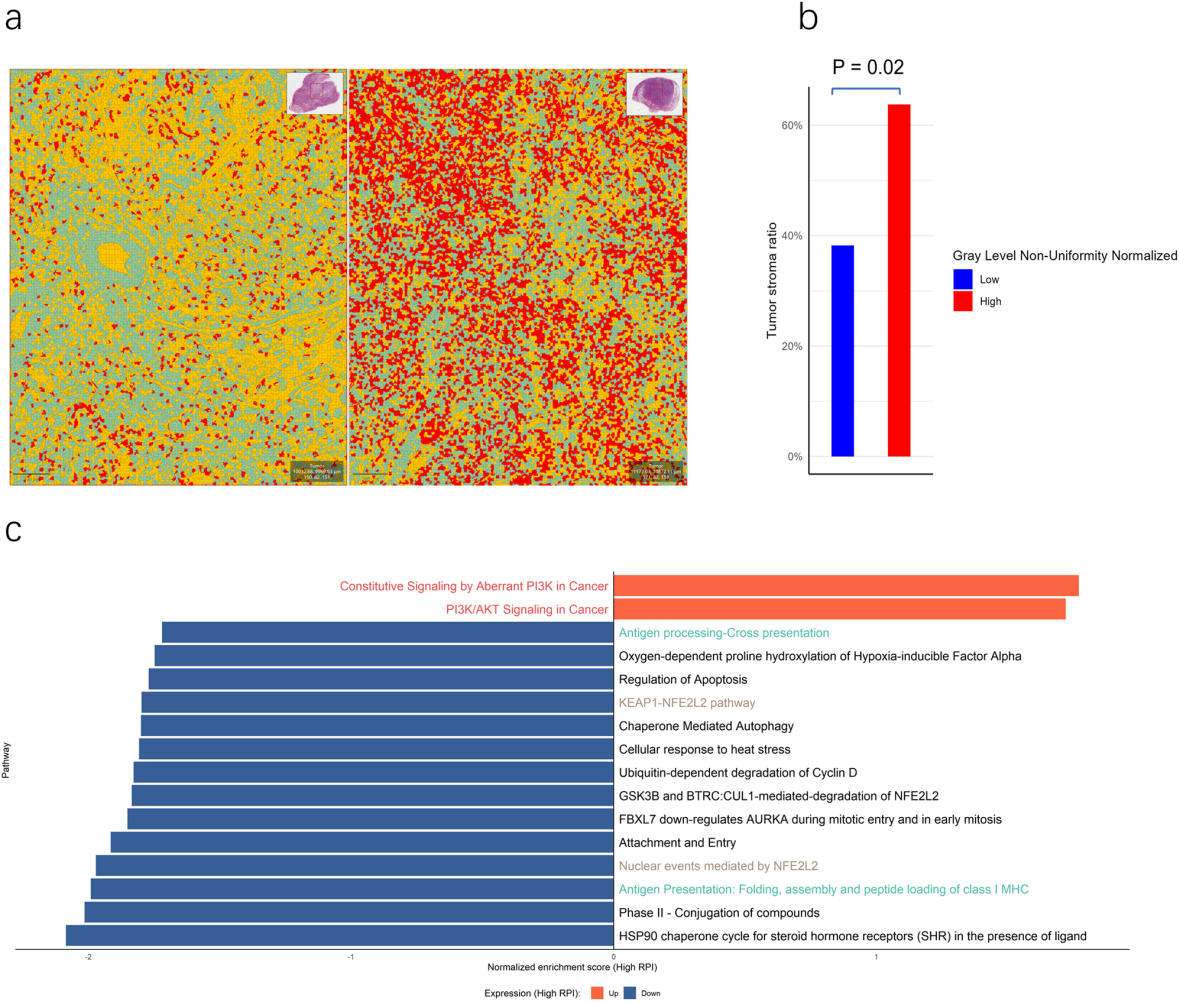
Pathological assessment and transcription analysis, and investigation of the underlying mechanism of the RPI. **a** Representative pathological slides highlighting the distinctive characteristics of tumours with low- and high-RPI values. Tumour regions are marked in red, stromal regions in green, and other components in yellow. **b** The tumour stroma ratio was significantly higher in the high GLNN group. **c** The Reactome pathways associated with RPI involved PI3K/AKT, KEAP1/NFE1L2, and antigen presentation. RPI, radiomic prognostic index; GLNN, Gray Level Non-Uniformity Normalised

We explored the biological basis of the RPI through pathological evaluation and transcriptomic analysis. In pathological evaluation, we found that the Gray Level Non-Uniformity Normalised (GLNN), one of the three selected radiomics features to construct RPI, was related to the TSR (Spearman correlation coefficient = 0.227, *p* < 0.01). Representative pathological image tiles from both high and low GLNN groups were presented (Fig. 6a). Notably, patients with a high GLNN demonstrated a significantly elevated TSR compared to those with a low GLNN (*p* = 0.02, Fig. 6b). Differential gene expression analysis between the high- and low-RPI groups were shown as volcano plots (sFigure 3a); top 10 up- and down-regulated genes are shown in sFigure 3b. The Reactome pathways associated with RPI involved PI3K/AKT, KEAP1/NFE1L2, and antigen presentation (Fig. 6c). The KEGG GSEA analysis also highlighted that the RPI was related to immune system response, especially antigen presentation (sFigure 3c).

## Discussion

The utility of PORT for patients with NSCLC is disputed [1–4]. While retrospective studies suggest PORT’s benefits [5, 6], recent RCTs [7, 8] demonstrated non-significant improvement in DFS or OS from PORT for unselected patients with NSCLC, albeit with a significant reduction in locoregional recurrence. Consequently, the focus has shifted to identifying high-risk individuals who might benefit from PORT. Prior studies revealed that PORT could enhance OS in carefully selected patients; however, these studies were based solely on clinical features and lacked external validation [13, 14]. Radiomics, which use quantitative imaging characteristics, has emerged as a promising tool for disease diagnosis and prognosis [22]. Our study showed that the RPI was a promising marker, and LRPI could identify patients who would benefit from PORT.

The LRPI is the first radiomics-based index to predict the benefit from PORT in patients with NSCLC. Previous studies have explored the prognostic value of radiomic signatures in early-stage NSCLC and the benefits of chemotherapy. One study established a radiomic signature by applying a LASSO-Cox regression model to 13 radiomic features of 329 patients with stage I or II NSCLC to predict DFS and benefit from chemotherapy [23]. A comprehensive model combining radiomics and major clinicopathological characteristics improved the DFS estimate (C-index: 0.74) compared with the clinicopathological model alone (C-index: 0.71). Another study used LASSO-Cox with radiomics to categorise the OS risk of pathologic stage IA pure-solid NSCLC in 800 patients [24]. In both the internal and external validation sets, the radiomics signature for predicting the 5-year OS demonstrated an area under the curve of 0.78 and 0.75, respectively. In our study, five independent validation cohorts were used to evaluate the prognostic value of the RPI, and we verified the ability of the LRPI to predict the benefits of PORT.

This study calculated the LRPI to identify patients who could benefit from PORT. The moderate-risk LRPI group exhibited a significant improvement in OS. By contrast, PORT did not improve OS in the low-risk LRPI groups, indicating that administering potentially toxic PORT provided no additional benefit for the low-risk groups [25, 26]. For patients with high risk, the prognosis is often determined by controlling distant metastatic, rather than locoregional, disease management. In this context, a study revealed that PORT was beneficial for a specific patient group, characterised by a low risk of distant metastasis and high risk of locoregional recurrence [27]. This finding aligns with our observations, further underscoring the critical role of tailored therapeutic strategies in optimising patient outcomes.

In the multivariate analysis, RPI and PLN remained significant predictors for OS, whereas pT did not. The RPI may embody the characteristics of pT and histological information. Previous studies have shown that radiomic features can predict pT [28], histology [29], and grade [30] in NSCLC. PLN remained a significant variable in the multivariate analysis. The radiomic features were extracted from the tumour volume; thus, they could not represent information from the mediastinal lymph nodes. PLN has been reported to correlate with OS [31–33], and patients with high PLN have been reported to benefit from PORT [33, 34]. In addition, studies have shown that the lymph nodes’ phenotypic information enhances the primary tumour’s performance for predicting the pathological response [35] and progression-free survival [36] in NSCLC.

To include the RPI in a clinical model, we developed an LRPI that combined the RPI with PLN based on the results that PLN and RPIs remained significant predictors of OS in the multivariate analysis. In predicting OS, our model outperformed the pT stage and clinical model, including the pT and PLN. Previous studies have shown that radiomic features can predict pT stage [28], histology [29], pathological grade [30], and lymph vesicular invasion [37] in NSCLC. The RPI could represent more information than the clinicopathological variables; thus, it would be much more convenient to use the RPI as it can be fully automatically calculated from the CT image. In contrast, clinicopathological variables require experienced doctors to identify [38]. Additionally, we verified the potential of an LRPI to predict the benefits of PORT.

We identified a relationship between GLNN and TSR. GLNN measures the similarity in intensity values across the image, with a lower GLNN value indicating a greater homogeneity or uniformity of intensities. CT images provide density information of the tumour, which may vary along with the TSR. A higher TSR indicated a more hostile tumour microenvironment, often associated with poorer outcomes [39]. Besides its relationship with TSR, GLNN may also reflect intratumor heterogeneity. The structural diversity is linked to a varying blood supply within the tumour [40], leading to a hypoxic environmessociated with a less effective response to radiotherapy in NSCLC [41, 42].

The Reactome pathways associated with RPI involved PI3K/AKT, KEAP1/NFE1L2, and antigen presentation. The PI3K/AKT and KEAP1/NFE1L2 pathways are associated with resistance to radiotherapy [43]. The PI3K/AKT pathway has been strongly linked to both initiation and progression in NSCLC [44, 45]. In contrast, the KEAP1/NFE2L2 pathway is involved in managing cellular stress and can shield cancer cells from the impact of radiation therapy [46, 47]. Moreover, KEGG GSEA analysis consistently demonstrated that RPI was related to immune system processes, including antigen presentation. The prominence of immune system-related functions suggests that cancer cells might escape host immune surveillance, underscoring the potential of immunotherapies. With the release of encouraging results from clinical trials involving the administration of immunotherapy to patients with peri-operative NSCLC [48, 49], a novel adjuvant treatment approach has emerged. Immunotherapy demonstrates substantial potential to enhance long-term survival rates for patients with operable lung cancer. Future research should therefore further explore these biological processes as potential therapeutic targets for preventing NSCLC recurrence.

Our study had several limitations. First, our study follows a retrospective design, inherently prone to selection bias that could not be completely mitigated. Despite the utilisation of participants from the PORT-C RCT, it is crucial to acknowledge that preoperative CT scans were limited to a subset of the overall cohort. Consequently, this inclusion constraint may introduce inherent biases that warrant consideration when interpreting the study results. Second, we restricted the reconstruction kernels and slice thicknesses to maintain the reproducibility of radiomic features, thereby likely limiting the expansion of this model. However, our model showed satisfactory performance in the external cohorts, which had a variety of scanners, kernels, and slice thicknesses. Last, external cohort 4 — comprising patients at varying disease stages who underwent definitive radiotherapy — may not precisely mirror the population composition observed in cohort 1. Nevertheless, validation of the RPI — initially developed from cohort 1 — was possible within this external cohort. This outcome underscores the robustness of the RPI as a stable prognostic variable to OS.

## Conclusion

We developed a preoperative CT-based RPI and an LRPI capable of predicting OS and benefit of PORT after curative resection of NSCLC. Additional validation at multiple sites is required to establish the RPI as a non-invasive biomarker for patient risk stratification, and as a predictive tool of PORT for NSCLC.

### Electronic supplementary material

Below is the link to the electronic supplementary material.

Supplementary Material 1

## Data Availability

The CT imaging data, clinical information, and whole slide images in cohort 1 and 2 are not publicly available for patient privacy purposes but are available from the corresponding authors upon reasonable request and for research only. The proposed source codes will be provided at GitHub. The CT imaging data, clinical information, and RNA sequencing data in cohort 4-6 are publicly available at The Cancer Imaging Archive (TCIA).

## Abbreviations

CT: Computed tomography
DFS: Disease-free survival
GLNN: Gray Level Non-Uniformity Normalized
GSEA: Gene Set Enrichment Analysis
KEGG: Kyoto Encyclopedia of Genes and Genomes
LASSO: Least absolute shrinkage and selection operator
LRPI: Lymph-radiomic prognostic index
NSCLC: Non-small cell lung cancer
OS: Overall survival
PLN: Positive lymph node
PORT: Postoperative radiotherapy
RCT: Randomised controlled trial
RPI: Radiomic prognostic index
TSR: Tumour stromal ratio
