# Supplementary material for: Preoperative CT-based radiomic prognostic index to predict the benefit of postoperative radiotherapy in patients with non-small cell lung cancer: a multicenter study
Source: Cancer Imaging. 2024 May 13;24:61. doi: 10.1186/s40644-024-00707-6 (PMC11089675; doi:10.1186/s40644-024-00707-6)

**Supplement tables**

sTable 1. Univariate Cox analysis of variables in non-PORT group of cohort 1

| Characteristics | HR.CI95 | P |
| --- | --- | --- |
| Sex (female vs male) | 0.77 (0.46-1.28) | 0.312 |
| Age (≤60 vs >60) | 1.38 (0.81-2.35) | 0.230 |
| KPS (≥90 vs <90) | 0.87 (0.51-1.47) | 0.606 |
| Tumor location (right lung vs left lung) | 1.28 (0.75-2.17) | 0.370 |
| Smoking history (presence vs absence) | 1.23 (0.74-2.03) | 0.430 |
| Histology (SCC vs non-SCC) | 0.68 (0.31-1.51) | 0.345 |
| pT (T2-3 vs T1) | 4.9 (1.53-15.65) | 0.007 |
| PLN | 1.06 (1.01-1.12) | 0.015 |
| Abbreviations: KPS, Karnofsky performances status; PLN: positive lymph node. | | |

sTable 2. Multivariate Cox analysis of variables possibly related to overall survival

| Characteristic | HR [95% CI] | p |
| --- | --- | --- |
| pT (T2-3 vs T1) | 1.36 [0.60, 3.06] | 0.461 |
| PLN | 1.10 [1.05, 1.16] | <0.001 |
| RPI | 2.03 [1.37, 3.02] | <0.001 |
| Abbreviations: PLN, positive lymph node; RPI, radiomic prognostic index. | | |
|  | | |

sTable 3. Patient Characteristics of Cohort 1

| Characteristic | Level | Non-PORT | PORT | p |
| --- | --- | --- | --- | --- |
| n |  | 132 | 96 |  |
| Sex (%) | Male | 69 (52.27) | 53 (55.21) | 0.76 |
|  | Female | 63 (47.73) | 43 (44.79) |  |
| Age (median [IQR]) |  | 56.00 [49.00, 60.00] | 55.00 [50.00, 60.00] | 0.73 |
| KPS (median [IQR]) |  | 90.00 [80.00, 90.00] | 90.00 [80.00, 90.00] | 0.96 |
| Smoking history | Absence | 79 (59.85) | 50 (52.08) | 0.30 |
|  | Presence | 53 (40.15) | 46 (47.92) |  |
| Tumor location | Left lung | 58 (43.94) | 34 (35.42) | 0.25 |
|  | Right lung | 74 (56.06) | 62 (64.58) |  |
| Histology | non-SCC | 114 (86.36) | 84 (87.50) | 0.96 |
|  | SCC | 18 (13.64) | 12 (12.50) |  |
| pT (%) | T1 | 27 (20.45) | 22 (22.92) | 0.78 |
|  | T2-3 | 105 (79.55) | 74 (77.08) |  |
| PLN (median [IQR]) |  | 4.00 [2.00, 6.00] | 4.50 [2.00, 7.00] | 0.30 |
| Abbreviations: KPS, Karnofsky performances status; SCC, squamous cell carcinoma; PLN: positive lymph node; IQR, interquartile range; PORT: postoperative radiotherapy. | | | | |

**Supplement figures**

**
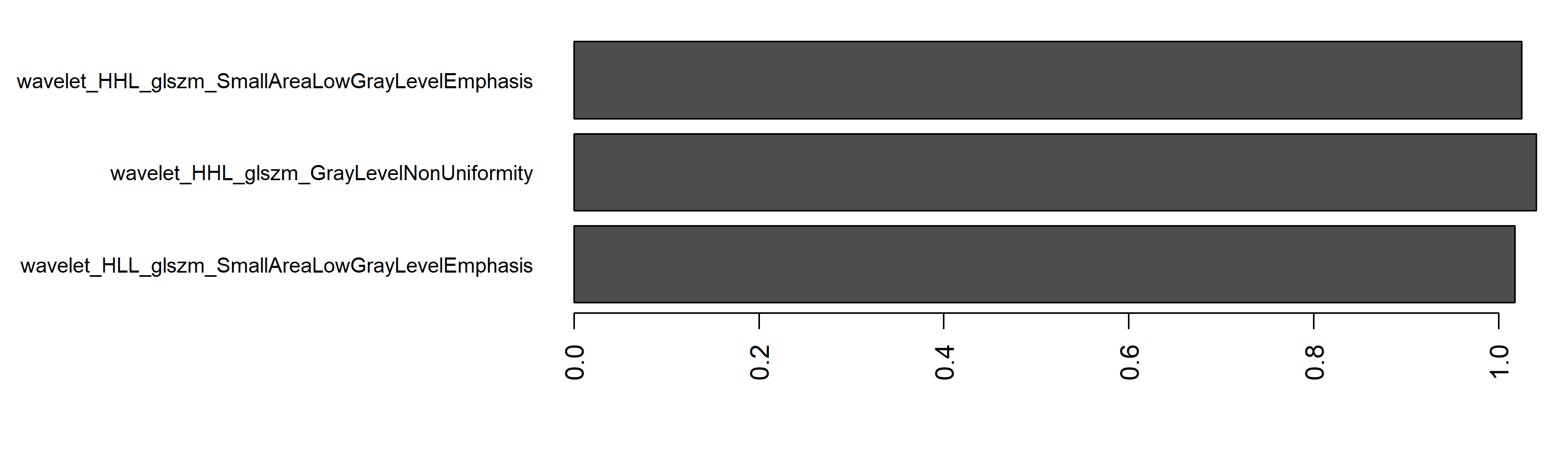
**

sFigure 1. LASSO Coefficients for top 3 selected radiomic features.

**
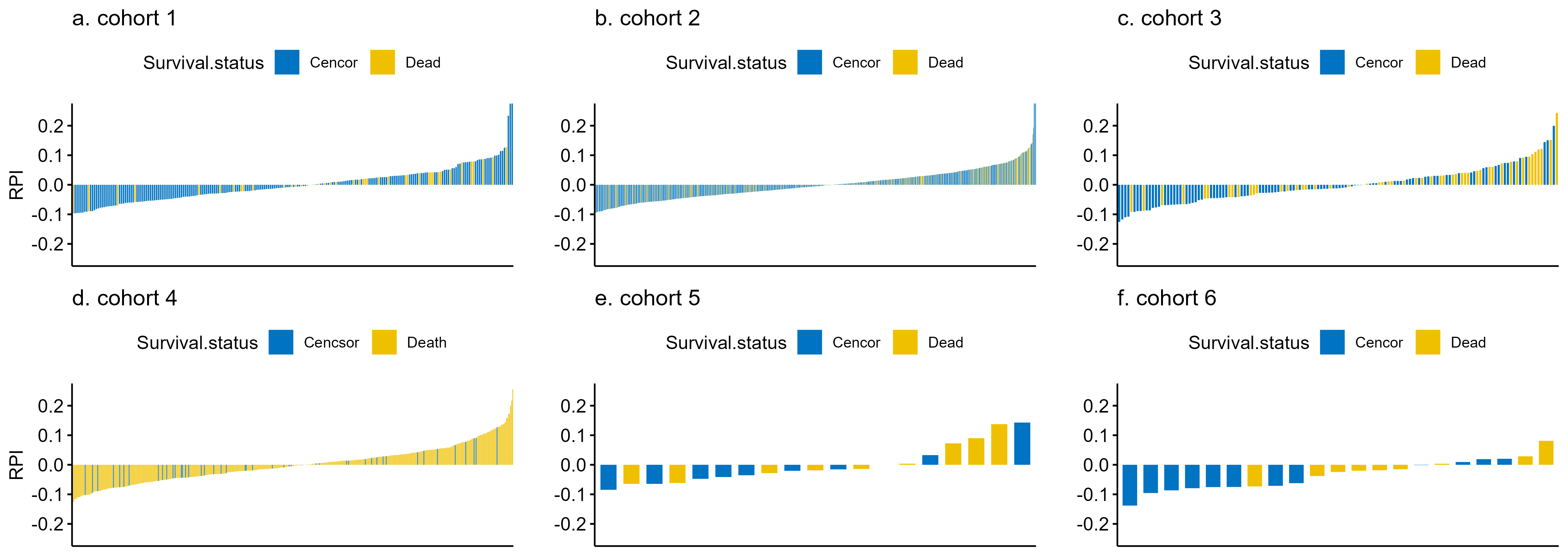
**

sFigure 2. Distribution of RPI across six cohorts. RPI, radiomic prognostic index.

**
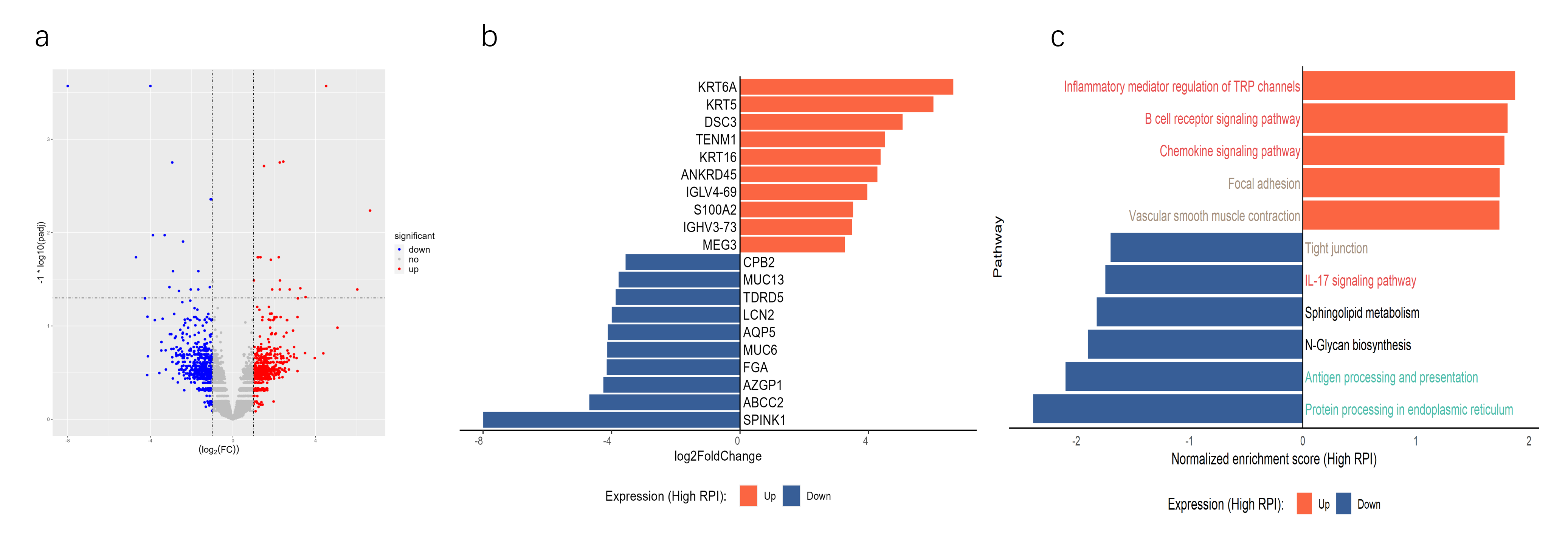
**

sFigure 3. a. Volcano plot: differential gene expression analysis between high- and low-RPI groups. b. Top ten genes with up- and down-regulation. c. The KEGG GSEA analysis highlighted that the RPI was related to immune system response, especially antigen presentation. RPI, radiomic prognostic index; KEGG GSEA, Kyoto Encyclopedia of Genes and Genomes gene set enrichment analysis.


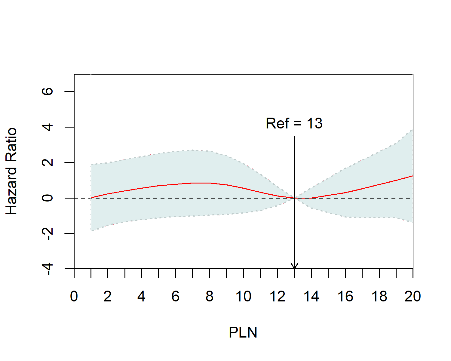


sFigure 4. Calculated logarithm of hazard ratios (solid lines), along with the 95% confidence intervals (shaded areas) for the association between PLN and overall survival.


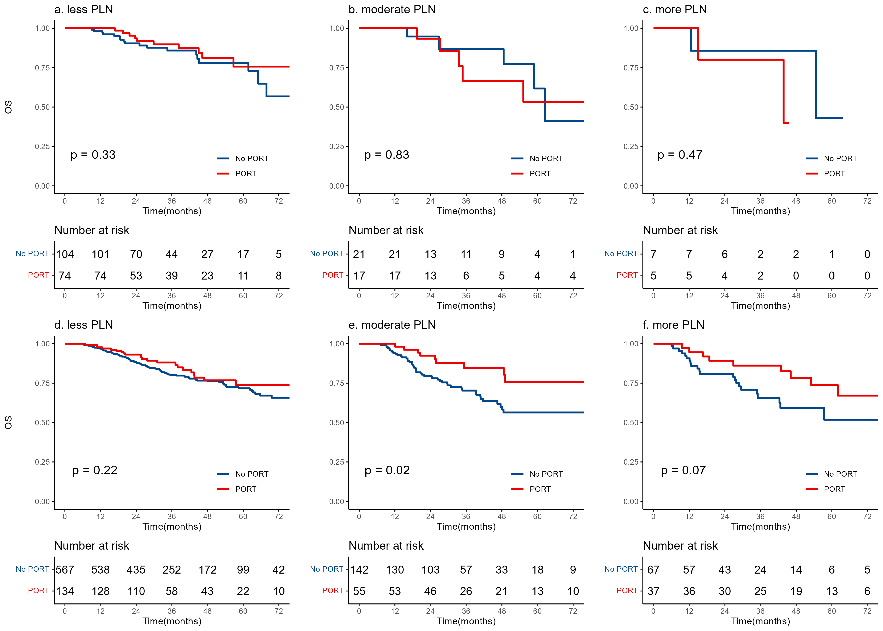


sFigure 5. Overall survival between PORT and non-PORT in PLN-based subgroups.

**Calculation of RPI**

The RPI was calculated using a linear combination of selected radiomic features, specifically:

RPI = 0.01740782*wavelet_HLL_glszm_SmallAreaLowGrayLevelEmphasis+ 0.04025639*wavelet_HHL_glszm_GrayLevelNonUniformity+

0.02483571*wavelet_HHL_glszm_SmallAreaLowGrayLevelEmphasis

**Supplement methods**

**Calculation of tumor stromal ratio**

The digitized whole-slide images (WSIs) were acquired using a KF-PRO-020-HI pathologic scanner (Konfoong Bioinformation Tech), viewed at 40× magnification. The order of magnitude of pixels was 10^9^.

A proficient pathologist used QuPath's manual annotation tools to recognize and label specific tumor regions across 206 H&E-stained slides. We implemented a secondary script on all images, using QuPath’s SLIC superpixel segmentation command. This procedure subdivided each annotated region into 'superpixels' by employing a method known as simple linear iterative clustering. Moreover, this script calculated the average hue for each superpixel along with Haralick texture features from optical density values, utilizing QuPath’s "Add intensity features" command. We also executed QuPath’s "Add smoothed features" command, which computed a Gaussian-weighted sum of the characteristics of adjacent superpixels and added these to the existing attributes of each superpixel. This approach added further contextual data extending beyond the superpixel itself.

We selected a subset of 50 'training' images, which the pathologist used to manually train a random trees classifier. The classifier was trained to distinguish between different tissue areas such as tumor epithelium, stroma, and other categories (like whitespace, mucin, normal muscle, or necrosis). Throughout this process, QuPath used all available features to train the classifier as a background task, providing instant feedback on its performance.

Once we confirmed that the classifier was performing satisfactorily across the training images, we applied it to the entire image set. Subsequently, we exported the total area of superpixels assigned to each class. In the final step, we computed the Tumor Stromal Percentage (TSP) using the equation: TSP = (AS / (AE + AS)) * 100%. In this formula, AS represents the total area classified as stroma, and AE stands for the total area classified as epithelium.

**Definition and calculation of selected radiomic features**

1. **Gray Level Non-Uniformity Normalized (GLNN)**

GLNN measures the variability of gray-level intensity values in the image, with a lower value indicating a greater similarity in intensity values.


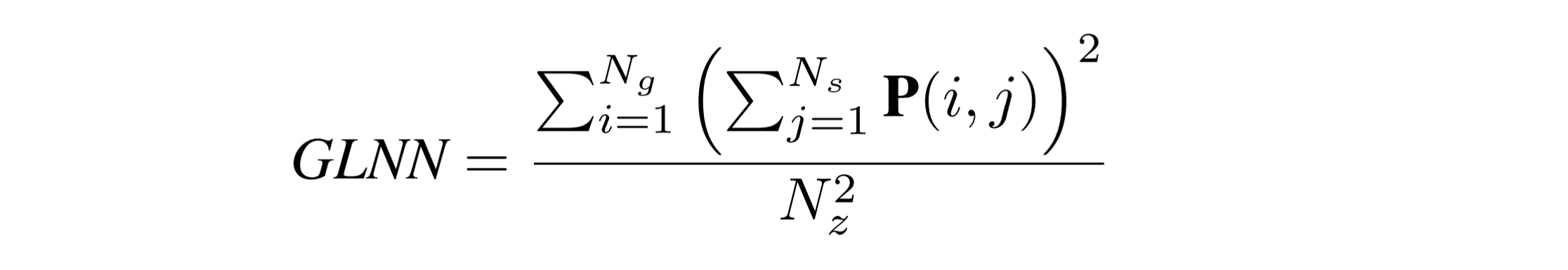


1. **Small Area Low Gray Level Emphasis (SALGLE)**

SALGLE measures the proportion in the image of the joint distribution of smaller size zones with lower gray-level values.


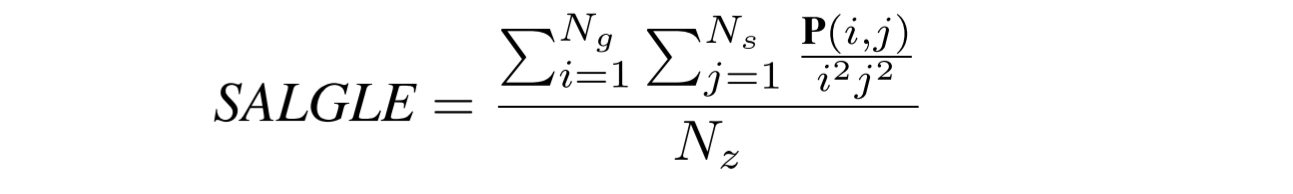

Supplement: Supplementary file 1 — Supplementary Material 1 [file 40644_2024_707_MOESM1_ESM.docx]
